# EFFECTS OF HEARING INTERVENTION ON PHYSICAL ACTIVITY AND FUNCTION: FINDINGS OF THE ACHIEVE RANDOMIZED TRIAL

**DOI:** 10.1093/geroni/igae098.0608

**Published:** 2024-12-31

**Authors:** Alison Huang, Jennifer Schrack, Nancy Glynn

**Affiliations:** Chair; Co-Chair; Discussant

## Abstract

This session examines the long-term effect of hearing intervention on physical activity and function. The Aging and Cognitive Health Evaluation in Elders (ACHIEVE) randomized controlled trial (2018-2022) is a multi-center randomized controlled trial of 977 adults aged 70-84 with untreated hearing loss and without substantial cognitive impairment (Clinicaltrials.gov Identifier: NCT03243422). The trial’s primary outcome was to determine efficacy of hearing intervention for reducing 3-year cognitive decline. Data on exploratory outcomes, such as physical activity and function were also gathered. Associations between hearing loss and lower physical activity and function have been consistently demonstrated across large epidemiologic studies. The mediating factors that may underlie these associations include changes in auditory awareness, communicative difficulty, cognitive decline, and/or reduced social connection. Hearing intervention (e.g., provision of hearing aids and related technologies, counseling, and education) may modify these mechanistic pathways and could potentially serve as an intervention for improving or maintaining physical activity and function among older adults with hearing loss, but has yet to be investigated in a randomized controlled trial. This session will describe the overall study design of the ACHIEVE trial and present results on the 3-year effect of the hearing intervention vs. health education control on four exploratory outcomes of physical activity and function: accelerometry-based and self-reported physical activity, fatigue, and physical performance.

